# Global discourses and experiential speculation: Secondary and tertiary graduate Malawians dissect the HIV/AIDS epidemic

**DOI:** 10.1186/1758-2652-14-47

**Published:** 2011-10-04

**Authors:** Tyler W Myroniuk

**Affiliations:** 1 Department of Sociology, University of Maryland, College Park, MD, USA

## Abstract

**Background:**

Since the beginning of the HIV/AIDS epidemic, the perspectives of secondary and tertiary school graduates in sub-Saharan Africa regarding the effectiveness of government and international HIV/AIDS policies and programmes have not been thoroughly examined. When extensive monetary aid is directed toward "development" in a country like Malawi, it is the educated elites - secondary and tertiary graduates who are heavily involved and influential in the domestic re-distribution and implementation of millions of dollars worth of aid - on whom international expectations fall to decrease the transmission of HIV. Many Malawian jobs related to public health and HIV/AIDS are created as a direct result of this funding and are occupied by the few secondary and tertiary graduates. Thus, it is a practical venture to understand their perspectives on highly contentious and heavily funded HIV/AIDS issues that affect their nation.

**Methods:**

Qualitative data was collected in this study in efforts to discover in-depth perspectives on the HIV/AIDS epidemic. Thirty-eight secondary and tertiary graduate Malawians took part in semi-structured interviews. Data was analysed using an early grounded theory approach and subsequent themes of "global discourses" and "experiential knowledge of HIV/AIDS" emerged.

**Results:**

This group of Malawians frequently responded to questions regarding healthcare and access to medicine, sexual behaviours and methods of reducing the spread of HIV/AIDS by citing and explaining the widespread, international and "proper" responses. The secondary and tertiary graduate Malawians also discussed these same topics in terms of what they perceive or have experienced. Experiential responses, such as the counter-productivity of circumcision and condoms, the overestimation of HIV/AIDS prevalence, and calls for more authoritarian policing of commercial sex work, were remarkably divergent from the HIV/AIDS discourse.

**Conclusions:**

The opinions of this group of secondary and tertiary graduate Malawians do not always coincide with the current literature and policies. They give deeper insight into what is perceived and what may be taking place, and hint at what the future holds for their people. The widespread and divergent perspectives must be seriously considered because these experiences describe the potential positive and negative consequences that occur on the ground throughout Malawi as a result of HIV/AIDS policies.

## Background

Malawi, the self-proclaimed "warm heart of Africa", is a hot-bed for international aid and research. Malawi's adult HIV/AIDS prevalence is approximately 12% [1] and Malawi ranks 153^rd ^out of 169 countries on the Human Development Index [2]. Malawi's high HIV/AIDS prevalence and poverty, low socio-economic development levels, political, civil, and military stability, and public battle to minimize corruption make it an attractive sub-Saharan African nation for prospective donors and researchers. HIV/AIDS is a frequently discussed and highly contentious issue in this extremely poor nation. In 2008, Malawi received nearly US$1 billion in official development aid from Britain, Japan, USA, the International Monetary Fund, the World Bank and a host of other nations [3]. Apart from official aid, Malawi is appealing to non-governmental organizations (NGOs) and other donors due to a welcoming government.

Around 80% of Malawians live in rural areas, many of whom live in extreme poverty [4]. Much of the research conducted by international organizations, universities, independent researchers and domestic agencies in Malawi has focused on uneducated individuals, rural families, sexual networks, attitudes towards contraception, risky sexual behaviours, and how HIV/AIDS affects rural communities [5-10]. When extensive monetary aid for "development" comes to Malawi though, it is the educated elites - secondary and tertiary graduates who are heavily involved and influential in the domestic re-distribution and implementation of millions of dollars worth of aid - on whom international expectations fall.

Many Malawian jobs related to public health and HIV/AIDS are created as a direct result of this funding and virtually all of the respondents in this study had participated in various forms of HIV/AIDS work. Additionally, these educated elites are precariously situated on the edges of Malawian civil society. Nearly all of the secondary and tertiary graduates in this study have lived for extended periods in rural and urban areas. They have a much fuller understanding of their society as a result. However, the explicit perspectives of secondary and tertiary school graduates towards HIV/AIDS issues have been largely overlooked; this includes how they understand the epidemiology of HIV, what they think of the effectiveness of Malawi's policies and programmes to stem the epidemic, and their views of the consequences of high mortality due to AIDS for the future of their nation. Thus, it was a practical venture to understand secondary and tertiary graduate Malawians' perspectives on highly contentious issues that affect their society.

Bourdieu theorized that the ability to obtain cultural capital is closely linked to educational capital, which is measured by qualifications [11]. Bourdieu ultimately suggests that "higher-class" and more highly educated individuals are enabled "to maintain their class positions, and legitimate the dominant positions that they typically go on to hold" [12]. For secondary and tertiary graduate Malawians, it is a rational choice to learn the international discourses surrounding these issues in order to potentially obtain upward social mobility. Educated elites know that they must present "proper performances" of HIV/AIDS knowledge to improve their social statuses in efforts "to keep from moving downward" [13].

To fully understand the international HIV/AIDS discourse, one must be literate, understand English, and have the resources to stay up to date with recent developments. Secondary and tertiary education in Malawi provides the skills necessary to do so. Considering that only 15.5% of women and 26.3% of men reach secondary school [14], graduating from secondary school, entering a tertiary programme, and even completing tertiary education would seem quite uncommon. Thus, secondary and tertiary graduates are among a small, elite, relatively privileged and influential group within Malawi.

Swidler and Watkins' [15] research on aspiring or "interstitial" elites and national elites in Malawi makes it clear that the ability to speak English and discuss the jargon of the international aid community are two skills that instantly set these elites apart from other Malawians. These educated individuals act as the eyes, hands, ears and feet of donors as the interstitial elites implement (allegedly) sustainable programmes on the ground while the national elites become the "middlemen" and "brokers placed strategically at the intersection of international and national networks" [15]. The respondents in my study are similar to Swidler and Watkins' interstitial elites and akin to Dagnaud and Mehl's sub-elites who hold "cultural power" and have the ability to disseminate ideas, norms and values.

However, the sub-elites do not "run the bodies which draw up cultural policies, which decide what values are legitimate, which control information, which regulate the significant means of communication" [16]. Therefore and inevitably, the sub-elites in Malawi and much of sub-Saharan Africa must know their governments' and donors' discourses, especially regarding HIV/AIDS in current times, in order to maintain their status as sub-elites or move "upward". This does not mean that these elites will not challenge these dominant perspectives of the national and international elites.

Ferguson [17] observed the efforts of a group of young, educated elites, mostly composed of university graduates and low-level white collar workers, attempting to promote national cultural reform, a type of "renaissance", in Zambia. These educated elites had the networks, technical skills and knowledge of international affairs but, unfortunately, failed miserably. Their attempts were "at the mercy of overwhelming market forces and supra-national institutions", such as the International Monetary Fund. These (essentially) sub-elite Zambians were being styled as newly "responsible" African elites, but "had little or no control" [17].

In Namibia, the educated elites have played a precarious role as local and national leaders, but also strongly resisted governance and apartheid discourses. Educated elites in Namibia routinely promoted post- and anti-colonial discourses aimed at diminishing the effects of a colonial past, while at the same time, differentiating themselves from the rest of society as elites [18]. Luke and Watkins [19] found that following the 1994 United Nations International Conference on Population and Development in Cairo, national elites throughout Africa were enthusiastic but also resistant to western and donor ideals, as well as new HIV/AIDS policies. These highly educated elites embraced the rhetoric of improved public health policies for their nations, but were simultaneously opposed to the manner in which "donors pushed high-profile subjects, such as HIV/AIDS, regardless of the country's own priorities". These same mixed-feelings are present today among those who are aware of or actively engaged with the international, academic rhetoric and discourses on health policies in the developing world.

The identities and aspirations of highly educated people in sub-Saharan Africa are inevitably tied to dominant global institutions and discourses. Since the educated elites' lives frequently intersect with the demands of the rest of the world (especially as "development" and HIV/AIDS projects backed by millions of US dollars enter sub-Saharan Africa), analyzing the opinions of these elites with respect to this reality is crucial.

I will shed light on how competing discourses surrounding HIV/AIDS and health policies are articulated by a group of secondary and tertiary graduate Malawians. They frequently responded to questions regarding healthcare and access to medicine, sexual behaviours and methods of reducing the spread of HIV/AIDS by citing and explaining the dominant, international and "proper" responses. The secondary and tertiary graduate Malawians also discussed these same topics in terms of what they perceive or have experienced.

Some responses were remarkably different and divergent from predominant HIV/AIDS discussions. Respondents appeared genuinely convinced of both global and experiential outlooks towards HIV/AIDS. To further understand the HIV/AIDS epidemic, not only in Malawi, but in sub-Saharan Africa, "experiential dialogue" concerning public health must be acknowledged and further examined. Divergent perspectives and knowledge may oppose global mandates or guidelines that shape the research agenda and determine (and disseminate) "valuable knowledge" [20]. Esacove [21] believes that this dilemma stems from the preference of western ideals in the Malawian "AIDS narrative". Experiential claims regarding HIV/AIDS by Malawians in this study may not always be empirically accurate, but "in the richness of [their] telling", make sense and are quite logical [21]. By understanding this dynamic that Esacove describes and by explicitly acknowledging and assessing the merits of divergent perspectives from the western AIDS narrative, policy can become more uniquely culturally oriented and the entire HIV/AIDS discourse broadened.

## Methods

Qualitative data were used in this study in efforts to facilitate the collection of in-depth and rich perspectives on the HIV/AIDS epidemic. Semi-structured interviews were conducted in order to help define social space and analyse "linguistic phenomena" that is often non-existent in quantitative survey data [22]. Respondents were asked questions regarding the strengths and weaknesses of public and private healthcare, access to antiretroviral (ARV) drugs, the perceived drivers of the HIV/AIDS epidemic, general government health policies, and the future of HIV/AIDS in Malawi. Minor revisions were made to the order of questions in the interview guide after several interviews in efforts to link these themes more adequately. Interviews took between 30 and 45 minutes each to conduct and were open to changes based on the interests and willingness of respondents. These interviews were audio-recorded (upon each respondent's consent) and then transcribed verbatim soon afterward.

NVivo 8 was used to help code the interviews into themes and sub-themes through an early grounded theory methodology (whereby new ideas and theories are generated through data analysis as opposed to formulating and testing theories prior to collecting data) so that new ideas, insightful commentary and in-depth language analysis could take place [23]. Moreover, since many potential secondary and tertiary graduate respondents were employed by NGOs, worked in the public health system, or attended tertiary schools where HIV/AIDS is frequently discussed, I believed that they would be adept in expressing their views on the topic.

A grounded theory methodology allowed respondents' ideas to flourish as this group provided a great "theoretical relevance" in eventually developing emerging categories because of their extensive experiences within the realm of HIV/AIDS [22]. Thus, respondents were allowed to express their views more freely in the interview setting whereas a design attempting to confirm a hypothesis may have been constricting. Two of the main themes, "global discourses" and "experiential knowledge of HIV/AIDS", and their respective subthemes emerged in data coding and will be examined in this study.

### Sample and study sites

Research was conducted in six districts throughout rural and urban Malawi: Rumphi in the northern region, Mchinji in the central region, and Balaka, Machinga, Zomba and Blantyre in the southern region. The locations of interviews were based on the respondents' preferences and took place in a variety of settings: the sides of dirt roads, meeting rooms, hallways, motel rooms and courtyards. From May to August 2010, 27 men and 11 women between the ages of 18 and 35 (inclusive) took part in semi-structured interviews with me. No other individuals were present in the interview setting. Due to my inexperience with vernacular languages, such as chiChewa, chiTumbuka, and chiYao, I conducted the interviews in English. The vernacular languages do not have equivalents for many medical or HIV/AIDS policy terms so when a Malawian speaks to another Malawian about these issues, a combination of vernacular and English is used. This creates an immeasurably different linguistic exchange than one solely in English, which is a second language for all of the respondents. The Malawians who participated in my research all graduated from secondary school and acquired their Malawi Schools Certificate of Education; many had tertiary training, ranging from trade certificates to university degrees, in progress or completed.

Prior to entering Malawi, I had no contacts or a group of willing respondents for the interviews. I used a combination of snowball and convenience sampling out of sheer practicality to find participants. By no means is this sample representative of all Malawians; nor was I trying to achieve such a thing. However, I managed to interview a diverse group of secondary and tertiary graduates (see Table 1). Respondents were not told that they would receive compensation for their time at the end of the interview in order to minimize the unknown effects of an economically driven interview. Afterward, respondents were given MWK500 (Malawian Kwacha), or roughly US$3.33, enough to buy two meals at a local restaurant.

**Table 1:** Malawian sub-elite educational characteristics

Sex	Male	Female	Total
**Highest schooling attained**			

MSCE*	4	2	6

Diploma	6	-	6

Degree	17	9	26

**Tertiary programme**			

Accounting	4	-	4

Architecture	-	1	1

Liberal arts	4	4	8

Education	1	1	2

Science	1	-	1

Engineering	1	-	1

Technology	1	-	1

Theology	1	-	1

Marketing	1	-	1

Rural & community development	4	1	5

Medicine	3	1	4

Nursing	3	1	4

**Tertiary programme status**			

Complete	9	3	12

In progress	14	6	20

**Age (inclusive)**			

18 to 22	9	8	17

23 to 27	11	1	12

27 to 31	6	2	8

32 to 35	1	-	1

*MSCE - Malawi Schools Certificate of Education

### Limitations

As this is a qualitative study with a small sample size, there are several limitations. The data is not representative of all secondary and tertiary graduate Malawians, let alone all Africans. The results cannot be generalized due to the small sample and snowball methodology whereby social networks influence the characteristics of the data [24]. Also, as a foreigner, my presence impacted responses to an unknown degree. Respondents were made aware that I was a sociology graduate student during the consent process. I made every effort to indicate that I was an independent researcher, but some respondents also understood that I previously worked for an HIV/AIDS research-oriented NGO on an unrelated project and may have tried to answer questions based on what they think an NGO employee would like to hear.

As a researcher who did not have the chance to spend much time with respondents outside of the interview, who was possibly perceived as an NGO employee rather than an independent researcher, and as someone who does not speak any vernacular languages in Malawi, I was unable to verify the nature of respondents' opinions or whether or not they were in line with the genuine behaviours of respondents. In Goffman's terms, I was likely only privy to the front-stage performance of these respondents and cannot be certain if I actually was interviewing the actors backstage and accessing their genuine and "suppressed" opinions (Goffman 1959).

### Ethics

Ethical clearance for this study was obtained from the University of Alberta. All respondents were thoroughly informed of the nature of the study. Written consent was obtained from all but two respondents, who preferred to give their consent verbally. Respondents were assured of confidentiality and that pseudonyms would be used to keep their identities anonymous.

## Results

### Global discourses

All of the respondents referred to the most widespread and prevailing themes of prevention and treatment, ranging from the importance of condom usage, to the risks of concurrent sexual partnerships, to the demand for increased antiretroviral therapy (ART), in recent African HIV/AIDS policy and literature. Respondents indicated that their knowledge of such issues came from secondary and tertiary classes, newspapers, television, radio and through NGOs and community-based organizations. Discussions of the dominant perspectives on how to reduce the spread of HIV/AIDS in Malawi (and also Africa) were filled with policy references, jargon, research examples and donor preferences. These sub-elite Malawians certainly knew the AIDS narrative and were more than willing to articulate their knowledge of the epidemic in detail.

In much of sub-Saharan Africa, and especially Malawi, a large body of literature focuses on concurrent sexual partnerships and the resulting "webs" of HIV/AIDS transmission. Concurrency is a relatively new but convincing explanation for the relationship between sexual behaviour and the spread of HIV as it brings attention to overlapping sexual networks rather than simply the number of sexual partners an individual has. Migrant labourers, sex workers and polygamy are key contributors in the construction of sexual networks [25-28]. However, the relationship between multiple sexual partners and increased HIV/AIDS prevalence is not necessarily proven [29].

Polygamy was an inevitably popular topic in Malawi because in May 2010, the Malawian government announced that it was introducing legislation to make polygamy illegal, as unenforceable as it would be. Patricia Kaliati, the Minister of Gender, Children and Community Development, described this legislation as a move "to reduce gender based violence". She also claimed that "[Malawi] has HIV/AIDS and we need to protect our people" [30]. Approximately 25% of women in northern Malawi and 13% of women in southern Malawi were part of polygamous marriages in the mid-2000s, which indicates a decline in polygamy since the mid-1990s throughout the country [31].

Despite the declining popularity of polygamy, Kaliati's two main reasons for outlawing polygamy are in line with the nine priority areas of the Joint United Nations Programme on HIV/AIDS (UNAIDS) [29]: reducing sexual transmission of HIV; preventing maternal death and infant HIV infection; ensuring HIV treatment; preventing tuberculosis and HIV deaths; protecting drug users from HIV; removing legal and discriminatory practices that block responses to AIDS; stopping violence against women and girls; empowering young people; and enhancing social protection for people with HIV. This move by the Malawian government appeals to donor nations and institutions as polygamy has received heightened scrutiny in recent years to determine the effects of such relationships on both men and women, and the implications of sexual power imbalances [32,10,8].

The notion that transactional sex and sex work are large contributors to sexual networks and the spread of HIV/AIDS was heavily discussed as well. Especially in the context of Malawi and most of sub-Saharan Africa (where legislation protecting sex workers is minimal to non-existent), sex workers are "among the groups most heavily affected by the epidemic" [33].

Nearly the entire body of academic literature and international policy in sub-Saharan Africa suggests that condoms need to be promoted and used more frequently by those engaged in casual, marital and transactional sex [29,20]. These suggestions are supported by a variety of demographic, public health, sociological and medical researchers who have overwhelmingly shown the effectiveness of condoms in stopping the spread of HIV/AIDS and sexually transmitted infections [34-36]. The predominant scientific literature and policy suggestions of the United Nations (UN) and World Health Organization (WHO) also indicate that male circumcision is a crucial preventative factor in reducing the spread of HIV/AIDS. Clinical trials have shown that male circumcision can greatly reduce the chance of HIV infection [37-41].

In addition to suggestions that centre on behavioural change among sexually active individuals, a key scientific innovation in reducing the spread of HIV is in the prevention of mother to child transmission (PMTCT). With the proper antiretroviral treatment (ART) prior to giving birth, mothers who are HIV positive can virtually negate the risk of passing the virus onto their unborn child. The method has proven highly effective [42-44] and is widespread in Malawi. The Malawi National AIDS Commission reported a more than 50% increase in PMTCT service sites, as well as starting nearly 70,000 pregnant mothers on ART in 2008 [32]. This effort to promote PMTCT has been heavily publicized under the Malawian government's broader National HIV Testing and Counselling Week [45].

### Reiterating the AIDS rhetoric

Respondents often reiterated the global discourse and rhetoric while suggesting methods to decrease the transmission of HIV in Malawi. These responses were likely the result of their heavy exposure to the widespread views surrounding HIV/AIDS and the reality that many Malawian job opportunities are related to public health and HIV/AIDS due to international and domestic funding for survey research projects, public health outreach and HIV/AIDS civic education. These responses and potential solutions are scientifically validated and "correct" based on the hegemonic literature on HIV/AIDS in Malawi and Africa more generally.

### ABC: abstinence, be faithful, condoms

Respondents overwhelmingly said that by abstaining from sex, being faithful to your sexual partner, or using a condom if neither "A" or "B" could occur, were essential guidelines for Malawians to follow in order to discourage concurrent sexual partnerships and diminish the size of sexual networks. The ABC approach gained global popularity after Uganda adopted it explicitly as its national AIDS policy [46,47]. Respondents believed that this strategy was effectively adopted and conveyed by the Malawian government and NGOs, but also felt that it needed to be further emphasized to help rid Malawi of HIV/AIDS. Some respondents felt that A, B and C had varying degrees of importance as well:

Now the government stepped up with the introduction of the National AIDS Commission [NAC]. NAC enforces the ABC: abstinence, be faithful, use condoms. I think this strategy back then really helped a lot in spreading the message about prevention. (Simon)

But the best way is to abstain. That's the best way. And introduce condoms so that people should use them to not get HIV/AIDS ... But the best way is just to abstain. (Ibrahim)

I would make sure I would still work on abstinence - tell people to abstain. And the condom stuff - if you can't abstain, use a condom. (Love)

By acknowledging the usefulness of the ABC approach, the sub-elites identified the effectiveness of sexual barriers in decreasing the transmission of HIV through sexual networks. Many respondents articulated the same concerns about concurrent sexual partners and the possibility of increased HIV transmission.

### Decreasing the number of concurrent sexual partnerships

Respondents also felt that polygamy and transactional sex were key components to the spread of HIV/AIDS through concurrent sexual relationships. To prevent further HIV infections, they felt that behavioural change was required. When I asked respondents what they thought some of the effective policies that the government recently implemented in combating the virus were, they reinforced the government's stance and mentioned the need to discourage polygamy in efforts to reduce the overall spread of HIV/AIDS:

The Ministry of Gender now wants to seize out these kinds of polygamy ideas. So they're trying to say people should not be getting two to three wives. So there's going to be a law in Malawi - if it's going to be passed. So with those kinds of policies, at least the government is trying to see the loopholes. So they're trying to look at polygamy and seize out polygamy. If you're found with two wives, then you'll be in for it because you're entitled to spread HIV/AIDS. (Angus)

And there's this bill that one Member of Parliament wants to propose to say "There shouldn't be any polygamous marriages. Polygamous marriages should be outlawed in Malawi". Yeah, when you look at all those issues, both the government and NGOs have one goal, which is to make sure the prevalence of HIV/AIDS is reduced. (Balawala)

In the previous times, the Ngonis were able to marry maybe four, five wives ... That was normal. But this time around, the same Ngoni, if he or she tries to marry four wives, two wives, that is going to be looked at as if he has gone on an extreme ... Like maybe you say: "Why you do that? Don't you know of HIV/AIDS?" (Jeffrey)

One thing I think Malawi has to do to reinforce the issue of concurrent partners; try to reduce concurrent partners ... then [HIV/AIDS] goes around and you've got three partners and they've got three partners, so now there are nine partners, and they've got 81 partners. So you are in that web and it's very tough. What I've found is that in Malawi, most people have protected sex ... and then eventually people stop protecting themselves. But people that are having these kinds of partners are in long-term relationships. And if you're married and people also know you're married, the more trust you're building, the more you're inviting people into that web. So that area must be looked at critically and must be studied seriously because it really spreads HIV, I think. (PJ)

Similar to what the literature suggests, respondents identified a need for change in both individual and cultural behaviours. Reiterating such ideas confirmed that respondents recognized the call for preventative measures in order to stem the number of new HIV infections.

### Sensitizing the people

More generically, respondents indicated that more civic education and sensitization regarding the most common methods of HIV transmission were necessary to spread among the masses. Respondents portrayed civic education and sensitization as normatively beneficial in raising awareness and warning Malawians of the dangers of HIV/AIDS regardless of the medium or content of the message. More information, more radio broadcasts, more dramas and simply more discussion about HIV/AIDS in communities were assumed to combat the spread of HIV. This is not surprising given that donors' doctrines of sustainable development in Malawi and other African countries encourage locals and elites by "empowering them to take control of their own futures" with self-reliant community outreach efforts [15]:

Well, maybe the awareness campaigns ... they don't conduct many of those. They should do more on that area. Maybe the civic education should go to those remote areas and teach the people how it's spread and how it can affect them. (Linda)

Yeah, since the disease is already in Malawi, and a lot of people are aware that there is HIV/AIDS. The government has done a lot on the sensitization. There have been a lot of meetings. Of course, there have been a lot of programmes on the radio, the TV. On the part of sensitization, I think the government has done a lot. I can say that anywhere, every place in Malawi, people know that there is this disease. So on that part, the government has done good. (Jeffrey)

Well, on civic education, the NGOs have done quite a good job. They sensitize people on HIV and AIDS. And yeah, as far as I know ... they go tell people about HIV and AIDS, how it's spread ... (Bridget)

By informing Malawians of ABC, the potential health risks of concurrent sexual partnerships, and generally disseminating information about HIV/AIDS, respondents reiterated the global directive to increase awareness of HIV/AIDS and how to prevent further infections through various behavioural changes. In addition to prevention, respondents relayed the importance of treatment: the other widely acknowledged crucial element in the fight against HIV/AIDS.

### More antiretroviral drugs

Many respondents depicted the never-ending battle to "roll out" ARVs in order to treat HIV-positive individuals. Like many HIV/AIDS activists, clinical trial researchers and other academics, respondents felt that more ARVs are needed, in addition to increased access to ARVs in the country. The need for ARVs and benefits of the drugs are supported by governments, academics and NGOs. While respondents did not provide any manageable methods to acquire and provide more ARVs to Malawians, their faith that the drugs are necessary in the battle against HIV/AIDS was unwavering:

What would I do if I was President? That one will be hard ... if I [only] had six months. Maybe I will just provide more ARVs to the people. (Linda)

As for me, the first policy to me could be trying to localize the ARVs. In private institutions, people should be getting them and in rural areas, we should establish clinics where people go and get the ARVs free. (Angus)

When they're distributing the ARVs, it's like people can still live healthy while they have this HIV, yes. (Precious)

First of all, I would have the hospitals ... they should have enough medicines, enough ARVs. (Matthew)

But I think the only thing that I can urge the government to do is stuff where people don't have access ... to VCT [voluntary counselling and testing] and ARVs ... If they can reach out to them and have small health centres there, people could go there so they know what the whole thing is about. I think that would be very, very great. (Robin)

While the sub-elites called for greater ARV provisions, they acknowledged the current efforts made by the Malawian government to provide free or inexpensive ARVs to the people:

It's like with this government of Bingu wa Mutharika, they've put some policies in place where they bring in more ARVs ... honestly I'm not infected, but I just hear from the radio ... they are bringing in more NGOs and bringing in more ARVs in different private hospitals and government hospitals, mostly in government hospitals. So they are making sure there are more ARVs in government hospitals so that people access them. I think that's the only policy I've seen that the government has done well to help those that are infected. (Walije)

The government, especially this government from 2004 until now, that's when the free ARVs were introduced. Because at first you had to buy ARVs. That's when they introduced the free ARVs. You could just go to a hospital and receive ARVs for free. And government also distributes condoms in government hospitals for free. That is something the government has really done. And yeah, I think that's the major things they've done. (James)

Well, they have done something like introducing ARVs for those who cannot afford or pay money. It's commendable. I can say that they have done something special. (Winford)

These responses were in tune with NAC's treatment mandate to improve ART services throughout the country and to rapidly scale up patients on ART to "achieve the Universal Access target" recommended by the UN and WHO [48]. NAC has also noted that the survival rate of patients on ART has improved.

In addition to calling for more ARVs, respondents highlighted the importance of and need for increased PMTCT services:

When a woman is pregnant, when they're going for the antenatals [prenatal treatment], they're asked to come along with their husbands so they go for an HIV test. They're supposed to test them before delivery because they want the safety of the baby. If they're positive, there are some drugs that the women take so they're not transmitted to the unborn. So I think that one also is one that is being encouraged. (Darlene)

This government has introduced this programme which is called PMTCT. It's an abbreviation and it means prevention of mother to child transmission. It's another good development. It is done so that mothers who are HIV positive should not transmit the disease, the virus, to young ones. So it's one way of reducing the prevalence rate, especially among the young ones. (Maurice)

For no apparent reason though, progressive government policies, such as the National HIV Testing and Counselling Week and the implementation of community home-based care, were conspicuously under-discussed among respondents. These national programmes have displayed the Malawian government's concerted effort to reach out and provide services to combat HIV/AIDS and treat HIV/AIDS-infected citizens in remote areas of the country. In general, respondents rarely commented on government programmes and outreach. The silence that looms over these issues needs to be further probed to determine their effectiveness in the eyes of Malawian sub-elites.

### Experiential speculation and divergent perspectives from the HIV/AIDS discourse

While respondents clearly demonstrated their knowledge about dominant academic, scientific and policy-oriented viewpoints regarding the spread of HIV/AIDS and potential solutions to minimize new infections, many supported several less popular and often disregarded perspectives on the epidemic. These points of contention make for a heated debate on the value of scientific versus experiential evidence, who and what constitutes valid knowledge, and what to make of non-mainstream analyses of the HIV/AIDS epidemic. However, unlike in the discussions about the widespread perspectives about HIV/AIDS in Malawi and Africa, divergent views from the literature were predominantly speculatory and based on respondents' experiences.

These divergent outlooks towards HIV/AIDS posed newer, more critical questions of the hegemony of the entire HIV/AIDS discourse. The most highly contested areas surrounded the possibility that circumcision and condoms could further spread HIV. Also, respondents overwhelmingly felt that the prevalence of HIV/AIDS is significantly higher than current epidemiological estimates. Respondents also hypothesized the feasibility and effects of authoritarian policies, which have not been implemented in efforts to minimize the spread of HIV in Malawi: proposals that have not been actively acknowledged by the government or the international community. Not all respondents opposed the dominant discourse on these topics, but challenges to the literature surfaced and require further consideration. The discourse on HIV/AIDS prevention and assessment was therefore greatly challenged by many of the Malawian sub-elites in the sample.

### Circumcision

Some respondents strongly agreed with the merits of male circumcision that have been observed in clinical and practical settings. However a similar portion of respondents believed that male circumcision increases the chance and spread of HIV/AIDS. Respondents often associated circumcision with "risky cultural practices" that were performed by southern Malawians, often the Yao, where circumcision entails much more than just removing the foreskin. Circumcision is an initiation rite and is traditionally followed by unprotected sex. Ibrahim, a Yao respondent, indicated that young men are supposedly told to "try out their new look". Circumcision was viewed as a catalyst in increasing the spread of HIV. The World Health Organization's warning that "men who undergo circumcision should abstain from sexual activity for at least six weeks, or until surgical wounds are completely healed" [20], may not be culturally appropriate or possible to avoid for some individuals:

We have these cultural beliefs. I don't know how to express it in English. They take children when they are young to go for circumcision ... the counsellors who are facilitating this service, they tell young people to go for sexual intercourse soon after the circumcision. So with that behaviour, they promote young people to go for sexual intercourse, that is, unprotected sexual intercourse that promotes HIV/AIDS. (Lucius)

A boy is supposed to go for an initiation ceremony, where he is circumcised. When he is circumcised, they are told to experiment sex after that for them to feel that they are mature. So when they get out of that initiation ceremony, they are forced to get a girl so they can experiment sex as they have been instructed in the camp. (Charles)

Alright, I don't think there's much we can do, you can do to improve what's already happening. Because there are also these cultural practices which spread HIV, like polygamy and some practices, cultural practices done by some people - the Yao tribes, where I don't know the programme ... *jando *[male circumcision ceremony]? Yes, those kinds of stuffs. I heard that when the children are there, they ask them to sleep together so they learn how it is like when you're growing up and what they'll face. It's like they're encouraging sexual behaviours among the youths. It's not only boys who are involved in these practices - boys and girls both. But they have separate camps, so they'll take a guy from this camp and a girl from this camp and tell them to sleep together ... So if we can abolish these cultural practices, I think that will reduce HIV because there's always been talk that "this should be abolished, this should be abolished", but no action has really been taken. (James)

In 2006, the Malawi Human Rights Commission conducted 262 face-to-face interviews and 99 focus groups throughout the country, discussing a variety of topics related to cultural practices. When discussing male circumcision or *jando*, 17% of respondents claimed that the practice was highly prevalent in their area. The report describes the contextually risky behaviour associated with male circumcision: "Once the boys undergo circumcision they are considered mature and are actually advised to have sexual intercourse with any girl as soon as they go back home from *thedzo *[the initiation site]" [49].

Thus, getting circumcised does not automatically translate to "reduce your chance of HIV infection" in all Malawian contexts. In fact, some clinical research has shown that circumcision's effects on decreasing the spread of HIV are equivocal [50]. UNAIDS has stressed that the site of circumcision (clinical or traditional) does not matter as much as the safety of the procedure [37]. The reality is that in traditional settings, clinical safety, hygiene and even practitioners are unlikely to meet the standards that western medicine and global bodies require. The notion that circumcision in Malawi is a clinically proven method of diminishing the chance of HIV/AIDS infection can certainly be challenged until a more culturally inclusive and feasible approach is implemented.

### Condoms

Respondents were divided on the negative and positive health benefits of condom usage. While nearly all respondents described ABC (abstain, be faithful, use a condom) as an important strategy in reducing HIV/AIDS, uncertainty, scepticism and outright genuine disbelief of the scientific health benefits of condom usage arose. Respondents associated condoms with higher probabilities of sex, and since heterosexual sex is the primary method of HIV transmission in Malawi, increased sex would appear to lead to increased risk of HIV infection:

I feel like the presence of condoms ... to me, I feel like it's something that is still fuelling the spread of HIV/AIDS ... Because when I have a condom, you are assured of that "even if I can do it I will be safe". But say, for example, the one who has gone for drinking at a certain pub - you know, when somebody is drunk you always have false confidence - you even forget to use that thing yet you prepared by saying "I'm going to use this. I'm going to do it". Because you're drunk, you cannot be able to put on it properly. You may even forget or the lady you're sleeping with might not even remind you to put on the condom. So you are at high risk of contracting HIV/AIDS because you say "I have this condom". (Charles)

When they say ... "use condoms", it's like they're encouraging the people who do that. So instead of just saying "You have to abstain. Try your best to abstain", they say "Use a condom. When you're going out with your buddies, don't forget to take condoms with you". That is just like encouraging the spread of HIV because people say that "if I'm going to get a condom, I'm going to do it anyway. Why? Because I have a condom". But then condoms aren't 100% perfect - somehow, like 88% perfect. So the introduction of condoms wasn't a very good idea. (Precious)

I think if the government had done something like telling people that condoms are not 100% efficient ... But then they let people say if you can't hold yourself, then you have to use condoms. But then they're not emphasizing that condoms are not 100% efficient. People are opting for condoms and they are not told how to use the condoms ... But to my side, condoms are not 100% efficient and when they come, they have these boxes and so many cartons of condoms. They're just distributing condoms. I think it's encouraging people that "you can go on, you can go on and have sex". (Darlene)

I think these NGOs have to stop distributing condoms. When they distribute condoms to the villagers, the rate will increase ... They encourage sex. When they distribute, what they have to do is tell people "Abstain! Stop! Once you get married then you have to". You see? It's like "OK I have my own condoms. I'm going to find a girl and have sex with her". Maybe you get some feelings and you go there and there's some woman that tells you "I don't want a condom. If you want to have sex with me, leave the condom". Then you have gone already and you can't come back. (Ibrahim)

### Overestimation of HIV/AIDS prevalence

Current epidemiological estimates suggest that Malawi faces a 12% HIV/AIDS prevalence rate among adults aged 15 to 49 years [1]. Nearly all respondents greatly overestimated the current HIV/AIDS prevalence. HIV/AIDS prevalence could very well be higher than epidemiological estimates depending on the sample that actually gets tested.

Maybe I can say it's about 50%. They say it's reduced but I don't think so because you can see many people are educated and they know everything about AIDS, but they're getting infected. (Darlene)

I think now we're talking of 43% ... (Anna)

I'm not so sure, but it must be greater than 50% of the population; a lot of people have it. (Bridget)

Maybe 60-something percent. (Jane)

We can see that maybe ... we can assume 20% of the whole population having HIV/AIDS. (Jeffrey)

Presently, it is still quite good. I think it can be 46%. In 10 years, I think it will be 30%. (Laxon)

These estimates are based on experience and, generally, intangible measures. Anglewicz and Kohler [51] discovered that 95% of their respondents believed contracting HIV was highly likely even in one act of sexual intercourse with an infected partner. Due to the expansion of voluntary counselling and HIV/AIDS testing centres and Malawians' observations of their own and others' sexual behaviours, it seems apparent that one "must already be on the road to AIDS". This frightening prospect may act as a deterrent for Malawians to clarify whether or not they have HIV/AIDS and contribute to increased HIV transmission [52]. The high perceived prevalence of HIV/AIDS could reinforce this counterintuitive scenario.

Due to the high volume of discussion and public health and awareness campaigns, it is understandable why HIV/AIDS prevalence is estimated at much higher rates than what has been officially calculated. However, the certainty in which respondents answered the question regarding HIV/AIDS prevalence and the trend among these highly educated individuals to overestimate prevalence indicates that there is need for further examination and more expansive testing methods. If these respondents' experiential speculations are indeed more accurate than current epidemiological estimates, the Malawian government and international community would need to intervene quickly and on a large scale in order to prevent demographic collapse.

### Curtailing commercial sex work

Respondents routinely suggested that authoritarian methods related to heavier policing of commercial sex work could be effective in reducing the transmission of HIV. However, the idea that sex workers should be "locked up" and cleared from the streets readily opposes human rights discourse and may actually hinder HIV prevention efforts in sub-Saharan Africa and globally [53]. Also, such solutions would not remedy larger issues, such as poverty and gender inequality, two driving factors in the proliferation of commercial sex work among Malawian women [54].

Nonetheless, curtailing commercial sex work in Malawi follows a similar argument to outlawing polygamy, yet as a public health solution, has been left untouched by the government and donors in Malawi. If a practice that can increase HIV infection is legally enforceable rather than just nominally discouraged, then it may be justified. Many of the sub-elite Malawians claimed that the government and police needed to enforce stricter regulations in bars and on the streets to decrease commercial sex work, but also that the government had ultimately failed in policing their people to this point:

I think the government through the National Assembly has not passed some important bills. Like in the past, those who are commercial workers, I think were not supposed to be in the streets because they also play a role in increasing the transmission of the virus. So I think the National Assembly should have passed a bill to restrict those commercial workers so that they should not be loitering around the towns, in the streets, especially at night, because they play a part in increasing the transmission of the virus. (Maurice).

For example, you learn about prostitution. The government is doing nothing on this. Even in the constitution of the Republic of Malawi, there is nothing. The constitution is silent on that. You see the teenage girls, 12 or 13 [years old], who are prostituting. The government is looking at those girls but there is nothing that they are doing. If they could put at least a tough or very interesting regulation that the moment a girl is from about eight to 25, she is not supposed to be found in the bars where people go and they're drinking, maybe doing pressure things. If that could at least be implemented I feel this pandemic could be reduced. (Steven)

So if I was the President of Malawi, I would say that whichever girl is found at the bar - the ones we know are prostitutes, right? - cuff them! At first maybe you broadcast it on the radio saying, "Whatever prostitute is found at a bar, if she will be caught, she will get a 10-year sentence" and make sure everyone knows. And if you go around and meet them there, get them behind bars. But I'm not saying you can only get HIV from prostitutes. But I know where I come from, people go around with *mahules *[bar girls]. So I think if I was President of Malawi, that's one thing I'd do ... get them behind bars. (Robin)

Yet as a practice that increases the transmissibility of HIV in Malawi, there is certain logic presented by respondents to heavily repressing such practices through strong legislation and, unavoidably, excessive policing. In the realm of public health in sub-Saharan Africa, such direct and repressive suggestions have been left off the list of potential solutions to decrease the spread of HIV. Such authoritarian policies would still not be able to identify all women who are engaged in transactional sex and not easily identifiable as commercial sex workers.

## Discussion

While this data is not representative of all secondary and tertiary graduate Malawians, let alone sub-Saharan Africans, it is important to know how highly educated and elite individuals view and oppose the popular, international discourse on HIV/AIDS. The Malawians in this study laid out many elements of the global HIV/AIDS discourse with little prompting. However, the divergent perspectives from the literature - the possibly counterproductive nature of circumcision and condom usage along with a great overestimation of HIV/AIDS prevalence and implementation of authoritarian polices designed to curtail commercial sex work - by those who have read, heard and have been taught the widespread perspectives on HIV/AIDS cannot be written off as false, backward and imaginary.

These divergent views were rationally constructed based on respondents' experiences living in urban and rural areas in a country where HIV infection is highly prevalent. A closer examination of contentious elements between these widespread and divergent perspectives is crucial in continuing to develop effective HIV/AIDS policies within African nations and abroad. The views of the sub-elites in Malawi and sub-Saharan Africa must be seriously examined alongside villagers, headmen, urbanites, national elites, donors, academics and international organizations.

The sub-elites have educational capital and are connected to national and international networks that have the capacity to implement and change HIV/AIDS policies in Malawi. Like the Zambian educated elites that Ferguson describes though [17], these Malawians are implicitly controlled by higher ranking elites and international forces that may silence or disregard their opinions on the HIV/AIDS epidemic. As Swidler and Watkins argue, the "doctrine of sustainability" and overall will of international NGOs and donors dictates the livelihoods of the sub-elite and contradicts their promises of "autonomy, empowerment, self-reliance, and a coherent, rational modernity" [15] for the sub-elite.

However, the views of these highly educated elites still contribute to a better understanding of the HIV/AIDS epidemic and indicate that not all scientific and academic research and policy can authoritatively apply to populations in Africa. For academics, policy makers, international and national elites, and everyone else with a stake in decreasing the spread of HIV/AIDS, examining these views of the epidemic is the next step in decreasing HIV/AIDS transmission and allocating funding in more culturally appropriate ways.

A grounded theory methodology combined with a semi-structured interview process allowed for the discovery of unknown and previously unheard perspectives regarding HIV/AIDS prevention and treatment policies in addition to the global discourse. Thus, this research was not narrowly focused by hypothesis testing. In countries where HIV/AIDS prevalence is high and concerted multilateral efforts have not proven as effective as expected, giving an opportunity to those who understand the customs and cultures on the ground and have extensive knowledge of the international HIV/AIDS discourse to freely express themselves may prove useful in identifying incongruent elements of international policy and realistic domestic prevention efforts.

Listening to individuals at the grassroots level is not a new idea, but listening to those with the most complete understanding of the situation in the context of HIV/AIDS is innovative. Since Malawian sub-elites are going to interpret and implement donor initiatives and strategies in some form, it is important to know what experiential "filters" they use when accepting or rejecting the HIV/AIDS discourse on the ground. While their opinions may not always coincide with the current literature and policies, they give deeper insight into what is perceived, what may be taking place, and hint at what the future holds for their people. By building upon this approach, a paradigm shift in devising policies to combat HIV/AIDS could take place.

## Conclusions

The extensive array of HIV/AIDS discourse knowledge that these secondary and tertiary graduate Malawians displayed in the interviews is impressive. This indicates that the current, "modern" discourse on HIV/AIDS prevention is globally disseminating, for better or for worse. Esacove describes this discourse negatively and feels that divergence from it by Malawians is justifiable [21]. Esacove believes that the discourse forces sexual actors into being "modern" and that their decisions should be "autonomous, rational, and informed by fact-based knowledge". There are "mismatch[es] between Western narratives and logics and the Malawian context", which reinforce the idea that sexuality is a "site of formal sanctioned state control", and international control, too [21]. Divergence from the dominant literature of HIV/AIDS prevention, reduction and intervention among the secondary and tertiary graduate Malawians in this study emphasizes these points of contention. A more cooperative understanding between state, international and domestic actors will reduce tensions between global and experiential perspectives on the HIV/AIDS epidemic. New HIV/AIDS policies fostered by this shared understanding will likely be more effective in reducing HIV transmission.

## Competing interests

The author declares that he has no competing interests.

## Authors' contributions

TWM is responsible for writing and authorizing this manuscript.

## Authors' information

Tyler W Myroniuk is a PhD student in the Department of Sociology at the University of Maryland. He travelled to Malawi between May and August 2010 to conduct interviews for this article.
